# A Cross-Cultural Adaptation of the Czech Version of the Developmental Coordination Disorder Questionnaire: The Content Validity Part

**DOI:** 10.3390/children11040482

**Published:** 2024-04-18

**Authors:** Nikol Vlasakova, Martin Musalek, Ladislav Cepicka

**Affiliations:** 1 Faculty of Physical Education and Sport, Charles University, 160 00 Prague, Czech Republic; nikol.vlasakova@seznam.cz (N.V.); musalek@ftvs.cuni.cz (M.M.); 2 Faculty of Education, University of West Bohemia, 301 00 Pilsen, Czech Republic

**Keywords:** cross-cultural, translation questionnaire, developmental coordination disorder, DCDQ, motor disorder, Czech, validity

## Abstract

The Developmental Coordination Disorder Questionnaire (DCDQ) is a widely used parent questionnaire for screening motor coordination disorders in children aged 5–15 years. Despite increasing motor difficulties in children, a validated version is lacking in Central Europe. In addition, previous studies pointed out that several DCDQ items were shown to be problematic in different cultural environments. We found that the majority of these studies did not assess the item’s content validity approach for keeping the semantic form and linguistic intelligibility of the original items. Therefore, this study aimed to translate the DCDQ, determine the content validity of items, and adapt the DCDQ for Czech children aged 6–10 years, where the identification of motor difficulties is crucial. Back-translation was employed, and face validity was consulted with linguistic experts and occupational therapists. A sample of 25 bilingual parents and practitioners evaluated the translated version, with content validity assessed using the Content Validity Ratio coefficient (CVR). Initial CVR scores ranged from 0.6 to 1.0. Lower scores were found for items 14 and 15, which were shown to be problematic in previous studies. The reason for the lower content validity in these items was due to double negation. Following linguistic modifications, the CVR values improved (range: 0.87–1.0), indicating content and semantic stability. Our findings underscore the importance of considering content validity and language specificity, including issues like double negation, during cross-cultural questionnaire validation to mitigate potential psychometric concerns in the future. The adapted Czech version exhibits significant content validity, thereby warranting further validation of its psychometric properties.

## 1. Introduction

### 1.1. Developmental Coordination Disorder (DCD)

DCD is characterized by motor in-coordination, delayed gross and/or fine motor skills, as well as difficulty acquiring new motor skills [1,2]. DCD is defined as a condition marked by challenges in executing a variety of motor tasks, apparent from early childhood and frequently enduring into adulthood [3,4,5]. DCD is also a neurodevelopmental condition marked by impairments in the development of motor coordination, where there are differences in neural networks and brain activation patterns [6]. DCD impacts approximately 4 to 10% of children in the school-age range (American Psychiatric Association [7,8]. Over recent years, there has been growing recognition towards neurodevelopmental disorders, including DCD, leading to increased screening and diagnosis. This heightened awareness has resulted in more individuals seeking evaluation for motor coordination difficulties, potentially contributing to the perception that DCD is becoming more prevalent. Additionally, changes in societal factors, such as increased sedentary lifestyles and decreased opportunities for unstructured physical play, may impact motor development and contribute to the observed prevalence rates of DCD. Nevertheless, there are significant cultural differences in the frequency of children with DCD.

If motor difficulties such as DCD are not addressed and early intervention is not implemented, secondary problems occur in most cases. DCD primarily affects motor coordination skills, including gross and fine motor skills, hand–eye coordination, balance, posture, and motor planning. These children experience peer exclusion and limited social participation [9]. Research findings have suggested that social and emotional difficulties linked to DCD can manifest as early as preschool age [10,11]. As formal schooling commences, there is a notable surge in motor demands, amplifying the observable challenges faced by children with coordination issues [12,13]. Recognizing this condition in its early stages is deemed crucial to enable timely intervention, mitigating both the risks and secondary challenges associated with the disorder [14].

The prevalence of developmental coordination disorder (DCD) and its diagnostic procedures display some degree of variability. Some scholars argue that due to the complexity of existing tests and test batteries, the pursuit of a definitive “gold standard” for diagnosing DCD remains ongoing [15]. In recent times, numerous screening tools for DCD have been created [16,17]; among them is the Developmental Coordination Disorder Questionnaire (DCDQ’07) [18]. Although motor difficulties are increasingly common in European children [19], there is no validated version of the DCDQ in Central Europe. Currently, motor tests such as MABC-2, TGMD-2, and BOT-2 are used, but they are not easy to administer, require more time, and are staff-intensive. Applying the DCDQ questionnaire in a school environment for parents and teachers would enable rapid feedback with follow-up recommendations, diagnoses, or interventions. If DCDQ were to be implemented, it would be possible to detect the occurrence of certain deviations in motor development in a timely manner.

### 1.2. Developmental Coordination Disorder Questionnaire (DCDQ)

The Canadian English version of the DCDQ’07 [18] is a revised edition of the DCDQ [20] and is considered the most well-evaluated parental questionnaire for identifying DCD in children aged 5 to 15 [21]. Widely utilized for both research and clinical purposes, the DCDQ’07 is recognized for its validity, brevity, and accessibility [20]. Its strong psychometric properties have been consistently affirmed [18], and it has been recommended as a supplementary tool in DCD diagnosis [21]. Developed in Canada, the DCDQ is a standardized screening instrument designed for parents and teachers to detect DCD in children. Comprising 15 items, the questionnaire assesses motor difficulties across three factors: Control During Movement, Fine Motor and Handwriting, and General Coordination.

Control During Movement pertains to an individual’s capacity to regulate and coordinate movements effectively, encompassing precision, timing, and smoothness. Motor Skills encompass a broad range of movements involving muscle use and body coordination, categorized into fine motor skills (e.g., writing) and gross motor skills (e.g., walking). General Coordination Abilities refer to overall proficiency in coordinating various movements and tasks involving sensory integration, motor planning, and purposeful execution.

All 15 items of the DCDQ are tailored to the assessed age group, considering developmental milestones and age-appropriate activities. Replicable and user-friendly, the DCDQ is conducive to school environments, facilitating diagnosis and disorder assessment. Cross-cultural adaptations have yielded positive results in various countries, such as Germany [22], the Netherlands [23], Brazil [24], and Japan [25].

### 1.3. Validation of DCDQ in Different Environments

While the DCDQ has been validated in various countries, including Canada [26], Australia [23], and China [27], there is a need for validation studies in diverse populations to ensure its cross-cultural applicability. Montes validated the DCDQ in a Spanish-speaking population, providing evidence of its psychometric properties in this cultural petting [27]. Wilson validated the DCDQ in Canada, establishing its reliability and validity within this cultural context [26]. Schoemaker conducted a validation study of the DCDQ in Australia, providing evidence of its reliability and validity in this population [23]. Tseng conducted a validation study of the DCDQ in China, demonstrating its reliability and validity within the Chinese cultural context [27]. These studies contribute to the growing body of literature supporting the cross-cultural applicability of the DCDQ by validating its use in diverse populations around the World.

Cronbach’s alpha, a measure of internal consistency reliability, has been calculated across different validation studies: Wilson reported α = 0.94 in Canada [26], Schoemaker found α = 0.91 in Australia [23], and Garcia reported α = 0.89 in Spain [28]. These values indicate strong internal consistency across diverse cultural contexts.

In our Czech study, we conducted validated the DCDQ using a confirmatory factor analysis approach. The results indicated a good fit of the model and acceptable reliability of the Czech version of the DCDQ [29]. However, to achieve sufficient psychometric properties, we had to employ content validity. Despite being intended for publication as a priority, unfortunately, these findings were ultimately not published.

### 1.4. Cross-Cultural Difficulties

Although the Developmental Coordination Disorder Questionnaire (DCDQ) has been widely adopted in various countries, the majority of studies validating its use in different cultural environments have noted instances where certain items exhibited poor psychometric properties [24,27]. Consequently, researchers have been compelled to replace some of these items to enhance the modified version’s performance. Additionally, it has been observed that previous studies that implemented the DCDQ in diverse cultural and linguistic contexts failed to assess the content validity of its items, a crucial aspect of the cross-cultural validation process [30]. During cross-cultural validation, challenges may arise in the process of cross-cultural adaptation, and it is imperative to acknowledge the linguistic nuances that could impact the diagnostic accuracy of test items. Translating assessment measures for use in different cultural contexts requires careful consideration of cultural nuances to ensure the instrument’s validity. However, directly applying the definition and operationalization of constructs from one culture to another may overlook cultural differences in expression and comprehension, leading to a literal translation of standardized instruments that disregards the new cultural context [31].

Recognizing this, recent literature emphasizes the importance of a culturally sensitive approach to research and instrument translation, advocating for adaptations that consider unique cultural contexts. Consequently, when translating a questionnaire into a new language, the goal is to achieve equivalence while maintaining similarity to the source version and ensuring conceptual equivalence. This may require adaptations to the original version to capture the intended meaning accurately [32].

In some cases, items may be deleted from the translated questionnaire and the behavioral information may be modified from the item number diagnostic tool. The deletion may be affected by translation to a different language with different rules for intelligibility or understanding the semantic form of an item. Any alteration in wording or meaning during translation can disrupt this relationship, potentially impacting the reliability and validity of the questionnaire [33]. Therefore, overlooking the content validity assessment in the validation process is usually connected with semantic and linguistic intelligibility bias, leading to significant changes in items’ psychometric properties and resulting in the erroneous acceptance or rejection of hypotheses or entire theories [34].

Given these considerations and the importance of a clinical screening tool for developmental coordination disorder (DCD), the present study aimed to translate the DCDQ into Czech and ensure semantic and content accuracy for children aged 6–10 years. The ages 6 to 10 are critical for motor development due to rapid growth, acquisition of fundamental skills, participation in physical activities, importance for academic performance, and implications for overall health and well-being. This period represents a crucial window for promoting optimal motor development via structured activities and interventions. Our research is centered on the age bracket of 6 to 10 years, a pivotal period characterized by intensive diagnostic efforts targeting various aspects of child development, notably motor skills, within the Czech Republic. This age cohort undergoes comprehensive assessments that extend beyond motor functions, often leveraging significant support from educational institutions. Subsequent to evaluation, tailored intervention initiatives are deployed as warranted by assessment findings. This specific age range was selected for investigation owing to the pronounced diagnostic activities and tailored interventions prevalent within this demographic cohort.

Based on the aforementioned discussion, it is evident that the cross-cultural adaptation of the Developmental Coordination Disorder Questionnaire (DCDQ) has shown shortcomings in assessing the content validity of its items. This may account for the observed poor psychometric properties in validation studies conducted across different countries, resulting in varied estimations of the risk for the presence of developmental coordination disorder (DCD). Therefore, the objective of this study is to translate the DCDQ, evaluate the content validity of its items, and adapt the questionnaire for Czech children aged 6–10 years. We anticipate that due to linguistic disparities between Anglo-Saxon and Slavic languages, we will observe the lowest content validity and intelligibility in items containing double negatives, which will necessitate suitable modifications for the accurate operationalization of the DCD construct.

## 2. Materials and Methods

In the study, we expected to observe the same three-factor structure (Fine Motor Skills, Movement Coordination, and General Coordination) in the Czech version of the DCDQ questionnaire. Additionally, we anticipated that the responses between the original and Czech versions would not significantly differ in terms of statement equivalence. First, it was necessary to work with respondents who were fully proficient in both languages. Full competence was necessary to assess the content validity of the questionnaire. Seventy-eight bilingual parents were initially contacted for potential participation in the study. Of these, 63 responded affirmatively and were subsequently evaluated against additional criteria, including a demonstrated understanding of the research topic, proficiency in both languages and attainment of language proficiency level C1. Ultimately, 25 participants were identified who satisfied all specified criteria. Common reasons for exclusion among the remaining candidates included inadequate proficiency in English and insufficient familiarity with the subject matter. Respondents were successively presented with both versions of the questionnaire (first the original and then the translated version). The Faculty of Physical Education and Sports Ethics Committee approved the study and consent procedures No. 6/2021. Ethical approval from the school board and written consent from all participants were obtained before data collection began.

### 2.1. Procedure

The Developmental Coordination Disorder Questionnaire (DCDQ-07) necessitates parents to assess their children’s motor skills in routine daily activities, comparing them with peers of the same age. The questionnaire comprises 15 items, typically requiring an average of 10 to 15 min for comprehensive completion. These items are distributed across three subcategories, encompassing a spectrum of motor skills that are recognized as posing challenges for children with DCD. First, the ability to control movement; secondly, their fine motor skills (such as handwriting); and finally, the child’s general coordination abilities. Individual areas are measured on a five-point unipolar scale (from “not at all” to “extremely yes”). Each area is summed up to provide a total score. If a child receives higher total scores, this signifies better overall motor coordination [35].

The questionnaire has three age ranges (5 years–7 years and 11 months; 8 years–9 years and 11 months; and 10 years–14 years and 11 months) [35]. Translating questionnaires for intercultural use and research is burdened by methodological difficulties; this threatens the validity of the research [36]. However, some of these shortcomings are far more challenging to detect, leading to the incorrect conclusion that cultural characteristics are significant when, in reality, they branch from semantic deviations [36]. We characterize this translation process, as well as the Canadian version of the questionnaire, for the detection of developmental disorders in children [35].

### 2.2. Cross-Cultural Translation of the Questionnaire

The translation of the questionnaire followed the guidelines established for the cross-cultural adaptation of instruments [37]. This process encompassed translating the content into standard language, modifying cultural expressions and idioms, and, where necessary, entirely transforming certain items to convey the same concept in the target culture. In the first phase, two qualified independent translators with diverse backgrounds translated the questionnaire from English into Czech. The second phase involved synthesizing these translations into the Czech version of the DCDQ. For the third phase, two back-translations of the synthesis into English were performed by two independent native English speakers. In the fourth stage, an expert committee, comprising the original translators and an occupational therapist, finalized the questionnaire, ensuring semantic, idiomatic, experiential, and conceptual equivalence.

Face validity was rigorously evaluated with a panel of independent linguistic experts from Charles University in Prague, renowned for their specialized proficiency in language translation and psychometrics. Additionally, invaluable insights and feedback were sought from highly experienced occupational therapists specializing in the assessment and treatment of motor coordination disorders in children. Moreover, the input of professionals from a distinguished educational–psychological counseling center in Pilsen, recognized for their expertise in child development and psychological assessment, further enriched the validation process.

### 2.3. Assessment of the Preliminary Version and Preparation of the Test Version

The back-translation method is commonly recommended for cross-cultural translation of questionnaires. In this approach, the questionnaire is translated from the original language to the target language by two independent translators, typically referred to as Translator A and Translator B. This helps eliminate potential inaccuracies and ensures a more reliable translation.

In the process described by Valerand [37], four professional translators were involved. It was not explicitly stated whether Translators A and B worked simultaneously or independently, meaning that Translator A translated the questionnaire without consulting Translator B, and vice versa. Once Translators A and B completed their translations independently, their translations were compared and reconciled to identify any discrepancies or differences. Any inconsistencies were discussed and resolved to produce a final version of the translation that accurately captured the intended meaning of the questionnaire in the target language. This process helps ensure the accuracy and validity of the translated questionnaire by incorporating multiple perspectives and minimizing the risk of errors or misunderstandings that could arise from a single translator’s work.

An important point of attention is the absolute preservation of the meaning of the items. After the translation was completed, the two translated versions of the questionnaire were compared. After a joint discussion, the two translators had to agree on one version of the translation of the questionnaire that respected the grammatical and syntactical principles of the target language while preserving the meaning of the original version. This produced a final version of the target language translation and was then passed to the other two translators (C and D). They were then tasked to translate this version back into the original language. The second set of translators worked completely independently. However, it was necessary to keep the original version secret so that no influence/bias could be introduced.

In summary, while there is not a set number of translators required for cross-validation, employing at least two translators, utilizing back translation, and involving additional experts and quality control measures can enhance the reliability and validity of the translated questionnaire [38,39]. The specific approach may vary depending on the study’s characteristics and the languages involved. Our methodology was influenced by the guidelines proposed by Sousa and Rojjanasrirat [40] for translating the questionnaire. We also placed significant emphasis on selecting qualified translators in line with the International Test Commission guidelines for test adaptation [41] and Hambleton [42], ensuring a combination of language proficiency and cultural understanding.

In addition to the translators, we also involved n = 25 bilingual parents, who were responsible for eligibility and semantic comparison of the original and Czech versions.

### 2.4. Assessment of the Preliminary Version and Preparation of the Test Version

At this stage, the back-translation (by C and D) was assessed by comparing the differences between the original, standardized version and the preliminary version. This assessment was a task for an expert committee set up for this purpose. The committee was made up of the translators involved and a linguistic expert. When the meaning of the items in the back-translation version matched the original version, the translation was judged to be fully adequate and definitive. Logically, it is not possible to arrive at a literal correspondence between the back translation and the original version, but the meaning of the items was decisive. If inaccuracies were encountered, the target language translation had to be modified so that the meaning of the original statements was completely consistent with the meaning of the statements translated into the target language.

In the case of significantly problematic translation tasks, it is necessary to invite experts in the linguistic field to the committee to help achieve the correct wording with their expert commentary, which was not necessary in this case. We mainly dealt with the last two negative items. To uphold the coherence of the item, it was necessary to translate it in a manner that avoids double negatives, which are not customary in Czech and could be perplexing to respondents. However, to ensure logical clarity, the item had to be phrased positively, without negations. However, this modification would lead to the reversal of the item scale, which was not possible. So, we highlighted these critical items, and they need to be explained to respondents before they complete the questionnaire.

After the linguistic modifications, when the assessment of the committee was positive, the questionnaire could be considered a test version [41].

### 2.5. Content Validity

It was imperative to engage respondents who possessed full proficiency in both languages. Such competence was deemed essential for evaluating the content validity of the questionnaire. The initial ten respondents were sequentially presented with both versions of the questionnaire—first, the original version, followed by the translated rendition [43].

Content validity was assessed using the Content Validity Ratio (CVR) test. The calculation of CVR was performed utilizing the following formula:

$$CVR=\frac{n_{e}-N/2}{N/2}$$

N is the number of all experts, and n_e_ denotes the number of experts who marked the item as valid for representing the feature being measured.

The recommended critical CVR value, as suggested by Ayre and Scally, for assessing good content validity of an item is 0.8 or higher [44].

### 2.6. Questionnaire Corrections

Due to the General Data Protection Regulation (GDPR), adjustments were made to the questionnaire header. Additionally, modifications were made to the format of the scale. The meaning of the scale was preserved; however, a literal translation could not be employed as it would not have been comprehensible to the Czech population. According to feedback from a linguist and occupational therapist, the scale in the Czech translation was found to be misleading, a concern that was confirmed during the pilot study. While we did not need to replace certain items to enhance the adapted version, as others have done [24,27], some items were adjusted for improved semantic validity.

The description of the distance was kept only in meters, not in feet; this measure is not typical for the Czech environment. In question number 3, we omitted the term “birdie” and kept the ball, which substitutes this skill in the same way and is more typical for Czech culture. In the original version of the questionnaire, a clumsy child is compared to a “bull in a China shop”. However, this comparison is not typical for the Czech language and would not make sense to the respondent. Certain terms were adjusted slightly to accurately convey the intended meaning and ensure linguistic correctness, as literal translation failed to capture the intended meaning in the target language. The wording of the last two items in the questionnaire contained double negatives, diminishing clarity for respondents. As these questions are of the reverse type, they may cause slight confusion. To address this, the negative wording was bolded to visually signal the change in questioning to the respondent. Unfortunately, these reverse questions were positioned at the end of the questionnaire, which, in our view, is less than ideal since respondents may already be losing focus by that point. Therefore, we recommend informing the respondent in advance about this aspect of the questionnaire before completion.

The modified version was completed and rated by ten bilingual respondents. They completed a questionnaire and rated the relevance and clarity of each item on a four-point scale. A content validity index was computed for each item in the questionnaire. The critical CVR value for this number of respondents is 0.8, as recommended by Polit and Beck [45]. According to their translation, items with a CVR of 0.8 or higher can be considered evidence of good content validity. The individual questionnaire items exhibited a range of scores from 0.6 to 1.0.

## 3. Results

Table 1 presents socio-demographic data of participants and Table 2 presents the frequencies of responses for each item. Notably, items 4 (“Jump over”), 5 (“Run & stop”), 6 (“Plan activity”), 11 (“Like sports”), and 14 (“Elephant in a shop”) displayed a high frequency of responses in category five (extremely like your child). This implies that the child exhibits no motor clumsiness and adequately plans movement activities. Conversely, other items demonstrated varied response distributions, with a significantly higher frequency in response category four (a bit like your child). Across all questions, the frequencies of responses indicating motor difficulties, falling into categories one (not at all like your child) and two (a bit like your child), ranged from 0.1% to 2%.

The critical values were recorded for items 14 and 15, which are worded in double negation and are therefore harder for the Slavically spoken respondents to understand. Based on the results, the questionnaire was modified and completed by another group of 15 bilingual respondents. Critical items could not be modified due to the double negative. If modified to a positive sentence, the items would change the scoring results, and this was not desirable. A minor linguistic modification was implemented to enhance the comprehensibility of the questions. These items in the questionnaire were visually emphasized, and respondents were informed of this fact. The last two questions were thoroughly explained to the respondents. The results of the CVR indicate that the modified version outperforms the original, thereby demonstrating improved content and semantic stability.

## 4. Discussion

This study aimed to translate the DCDQ, determine the content validity of items, and adapt the DCDQ for Czech children aged 6–10 years, where the identification of motor difficulties is crucial. The DCDQ-CZ demonstrated equivalence with the DCDQ’07, a finding consistent with the Spanish version of the DCDQ [28].

We encountered challenges with double-negative statements in the questionnaire. To enhance clarity and prevent inconsistent responses, we revised the wording of these items, ensuring comprehension across all language proficiencies [46]. In the Czech adaptation of the DCDQ, problematic items 14 and 15 were identified due to double negation, making them difficult for Slavic-speaking respondents to understand. The researchers linguistically modified these items, resulting in improved content and semantic stability.German-speaking Countries: Kennedy-Behr [22] reported issues with double-negative wording in the German version of the DCDQ. While the Canadian French adaptation did not encounter such problems, similar challenges were documented in German translations. They conducted linguistic modifications to ensure clarity and consistency. Spanish Population: Montes-Montes [28] encountered issues with item clarity and comprehension in the Spanish version of the DCDQ. They conducted linguistic adjustments to enhance the readability and semantic equivalence of the questionnaire. These studies highlight the importance of identifying and addressing problematic items during the cross-cultural adaptation of the DCDQ to ensure its validity and reliability across diverse linguistic and cultural contexts.

However, altering critical double-negative items to positive statements would affect score outcomes, hence the importance of properly explaining their meaning to respondents. This is why these items are highlighted in the questionnaire.

It should be noted that the DCDQ is designed to capture a broader range of children compared to most normative standardized motor skill tests, serving as a “coarse sieve” to screen and identify potential difficulties [23]. It evaluates everyday skills through a questionnaire method, distinct from standardized motor tests, thus expecting a discrepancy in results between the two [35].

While several diagnostic tools focus on standardized and clinical testing to assess motor skill impairment, fewer tests evaluate the link between these difficulties and daily activities or school tasks. Parent reports have shown utility in diagnosing developmental and motor issues [47,48].

### 4.1. Limitations and Strengths

In numerous studies involving cross-cultural adaptation, content validity is often not assessed during the pilot phase, where adjustments to item clarity are still ongoing. Subsequently, the modified questionnaire is administered to a large respondent pool, compromising the validity of the results. In our study, we encountered challenges related to double negatives, a problem not reported in the Canadian French adaptation of the DCDQ’07 but observed in other cross-cultural adaptations, particularly those for German-speaking populations [22]. Rephrasing these statements aimed to ensure clarity across all language proficiencies, as ambiguity could lead to inconsistent responses [46].

One limitation of our study was the focus on a younger age group (6–10 years). However, a notable strength was the involvement of 25 bilingual parents from the field. Additionally, the translation process adhered to the rigorous protocol outlined by Beaton [36] and an even more stringent transcultural validation method as described by Vallerand [37].

### 4.2. Practical Clinical Implications

The results indicate that the DCDQ serves as a reliable and valid tool for evaluating motor coordination problems and identifying children with probable developmental coordination disorder (DCD) in the Czech context. Specific cut-off values, tailored to different age groups within the population, are provided for both research and clinical applications. This accessible and dependable measure facilitates a quick assessment of motor coordination, serving as a valuable initial step for a more in-depth evaluation of potential DCD when necessary. Healthcare professionals in pediatric primary care, including occupational and physiotherapists, as well as educational and psychological counseling staff, can utilize the DCDQ to operationalize the diagnostic criteria for DCD.

### 4.3. Future Research Implications

A recommendation for future research is to target the 11–15 age group, which was not addressed in this study. Further validation of the psychometric properties and potential standardization of the instrument can be pursued. The findings from this study support the feasibility of using the Czech version of the questionnaire and the prospect of standardization [29].

### 4.4. Study Conclusions

The DCDQ serves as a diagnostic tool for identifying motor difficulties and is intended to complement standardized tests. In recent years, it has gained recognition as a reliable assessment tool globally, particularly for evaluating activities of daily living as outlined in Criterion B. Via linguistic adjustments of items in the DCDQ that exhibited weak content validity in the Czech environment, it was possible to obtain a valid and reliable screening tool, the DCDQ-CZ 6-10, in the Czech environment.

However, it is important to note that the DCDQ alone should not be relied upon for diagnosing dyspraxia; instead, it is best utilized in conjunction with standardized tests [35]. Despite the adjustments made to the Czech version of the DCDQ during the validation process, this version can be considered semantically and conceptually equivalent to the original version.

In accordance with Rositer [34], we acknowledge that the assessment of content validity in the validation process is frequently linked with biases in semantic and linguistic intelligibility. These biases can result in significant alterations in the psychometric properties of items. Consequently, this may lead to the erroneous acceptance or rejection of hypotheses or entire theories.

## Figures and Tables

**Table 1:** Socio-demographic data of participants.

Participants			
		N₁ (10)	N₂ (15)
Gender (%)	Female	50	47
	Male	50	53
Age (%)	30–40	50	53
	41–50	40	33
	50–60	10	14
Bilingual (%)	CZ + EN	100	100
Czech nationality (%)		100	100

**Table 2:** CVR values for individual items of DCDQ-CZ.

	Modified DCDQ-CZ		Final Version DCDQ-CZ	
Items	Panel Size N₁	CRV₁	Panel Size N₂	CRV₂
1: Throw	10	1	15	1
2: Catches	10	1	15	1
3: Hits	10	1	15	1
4: Jumps	10	1	15	1
5:Runs	10	1	15	1
6: Plans	10	1	15	1
7: Writes fast	10	1	15	1
8: Writes legibly	10	1	15	1
9: Effort/pressure	10	1	15	1
10: Cuts	10	1	15	1
11: Likes sports	10	1	15	1
12: Learning new	10	1	15	1
13: Quick/competent	10	1	15	1
14: “Bull in china shop”	10	0.6	15	0.87
15: Not fatigue	10	0.8	15	0.87

## Data Availability

The data presented in this study are available on request from the corresponding author due to GDPR.
